# The American Medical Association—Thirty-First Annual Meeting

**Published:** 1880-06

**Authors:** 


### THE AMERICAN MEDICAL ASSOCIATION.—THIRTY-FIRST ANNUAL MEETING.

To New York city belongs the honor, the present year, of entertaining the members of the medical profession, gathered together in National Council to discuss important questions relating to medical science. The fact that the Association meets in the Metropolis, which is the recognized medical, as it is the commercial and financial centre, of the country, will attract hither large numbers, and it is safe to predict an unusually profitable and interesting meeting.

In the various sections, a large number of valuable papers will be read by the most eminent men in the profession, the discussions eliciting an active interest from those, who are fortunate to be able to lend an attentive ear.

The suggestion made by the *Medical Record*, that papers should be read in abstract, will, if heeded, give a wider scope and greater opportunities for ambitious writers, while it will lend additional interest and value to the proceedings.

The social entertainments given under the direction of the New York profession, will be on a scale of generous magnificence, we have no doubt, and will show the cosmopolitan character and ability of the men who now adorn the medical profession of that city.

We hope to gather from its proceedings valuable material for future numbers of the JOURNAL.

